# Robot-assisted ex vivo neobladder reconstruction: preliminary results of surgical skill evaluation

**DOI:** 10.1007/s11548-022-02712-1

**Published:** 2022-07-08

**Authors:** Ziyang Chen, Serenella Terlizzi, Tommaso Da Col, Aldo Marzullo, Michele Catellani, Giancarlo Ferrigno, Elena De Momi

**Affiliations:** 1 Department of Electronics, Information and Bioengineering, Politecnico di Milano, Milan, Italy; 2 Department of Mathematics and Computer Science, University of Calabria, Rende, Italy; 3 Istituto Europeo di Oncologia, Milan, Italy

**Keywords:** Surgical skill evaluation, Robot-assisted surgeries, Performance metrics, Significant differences

## Abstract

****Purpose**:**

Advanced developments in the medical field have gradually increased the public demand for surgical skill evaluation. However, this assessment always depends on the direct observation of experienced surgeons, which is time-consuming and variable. The introduction of robot-assisted surgery provides a new possibility for this evaluation paradigm. This paper aims at evaluating surgeon performance automatically with novel evaluation metrics based on different surgical data.

****Methods**:**

Urologists ($n=10$) from a hospital were requested to perform a simplified neobladder reconstruction on an ex vivo setup twice with different camera modalities ($n=2$) randomly. They were divided into novices and experts ($n=5$, respectively) according to their experience in robot-assisted surgeries. Different performance metrics ($n=2$) are proposed to achieve the surgical skill evaluation, considering both instruments and endoscope. Also, nonparametric tests are adopted to check if there are significant differences when evaluating surgeons performance.

****Results**:**

When grouping according to four stages of neobladder reconstruction, statistically significant differences can be appreciated in phase 1 ($p=0.0284$) and phase 2 ($p=0.01953$) with normalized time-related metrics and camera movement-related metrics, respectively. On the other hand, considering experience grouping shows that both metrics are able to highlight statistically significant differences between novice and expert performances in the control protocol. It also shows that the camera-related performance of experts is significantly different ($p=0.003153$) when handling the endoscope manually and when it is automatic.

****Conclusion**:**

Surgical skill evaluation, using the approach in this paper, can effectively measure surgical procedures of surgeons with different experience. Preliminary results demonstrate that different surgical data can be fully utilized to improve the reliability of surgical evaluation. It also demonstrates its versatility and potential in the quantitative assessment of various surgical operations.

## Introduction

The skill level of a surgeon, which is highly variable, can be determined by several factors, which include cognitive capabilities, judgment and decision making, and manual dexterity [1, 2]. With more recent advances in surgery, such as minimally invasive surgeries (MIS) and laparoscopy-based procedures, surgical technical skill requirements are increasingly more demanding due to the complexity of these procedures [3, 4]. Although significant strides have been taken to improve surgical technical skill training and assessments, methods to differentiate and standardize technical skill levels are still under development [5]. It is essential to emphasize the importance of surgical skill evaluation, because surgeons can conduct targeted practices based on the feedback obtained from the skill evaluation to improve their operation.

Manual skill assessment was widely adopted in previous stage, and it requires evaluators to manually rate performance. Generally, a senior surgeon observes a student performing a task and either gives verbal feedback or skill evaluation can be performed by using rated checklists [6]. It allows for greater standardization and better outcomes in terms of inter-rater reliability. However, it should be noted that manual assessment tools measure the evaluator’s perception of the surgical performance quality, which implies variability and unreliability of surgical evaluation criteria. Thanks to the development of robotic surgical assessment tools, it is currently available for quantitative skill evaluation [7, 8].

Hence, it is extremely important to find objective metrics that could appropriately describe the surgical performance without requiring the presence of an expert surgeon. One of the advantages of using surgical robots is the possibility to keep tracking movements made by the surgeon thanks to the tracking systems they have integrated, such as da Vinci system. This allows exploiting the other possible way of addressing surgical skill evaluation: automated assessment [9]. Different from the manual approach, this kind of evaluation is performed by the direct computation of quantitative performance metrics [10]. It means that during the surgery, robotic instrument kinematic tracking data (i.e., instrument traveling distance, moving velocity, acceleration and deceleration), system events data (i.e., camera movement, master clutch use, third instrument swap and energy application) and surgical video data (i.e., surgical footage annotation) can be recorded [7]. All this information can be successfully exploited to define objective metrics for surgical skill evaluation.

The aim of this work is to evaluate surgeon performance when carrying out an experiment conducted on the da Vinci Research Kit (dVRK) in a dry laboratory setup, comparing two ways of controlling the endoscope: automatically, through the implementation of System for Autonomous Camera Navigation (SCAN) [11] and manually (classical approach). Particularly, such analysis is carried out by defining appropriate performance metrics for surgical skill assessment, which serve to investigate the impact on the performance of different stages and surgeons’ experience.

## Methods

### System setup

As far as this experimental study is concerned, a standard first-generation dVRK, available at Politecnico di Milano, is used for data acquisition. The mechanical hardware can be divided into follower side and leader side [12]. For the follower side, two Patient Side Manipulators (PSMs), labeled as PSM1 and PSM2, are used to hold different surgical instruments, and an Endoscopic Camera Manipulator (ECM) is equipped with a full HD stereo endoscope. On the other hand, the leader side is essentially made up of the master console, composed of: two Master Tool Manipulators (MTMs), labeled as MTML and MTMR, aim for teleoperation; a High-Resolution Stereo Viewer (HRSV) with a resolution of $640 \times 480$ can provide 3D surgical scenes for the surgeon; and a foot-pedal tray is used to switch between different operation modes.

Two different control modes of the endoscope are adopted to conduct the experimental study, one is the manual control by surgeons and another one represents the autonomous endoscope navigation which is achieved by the SCAN algorithm in [11] to provide the user with an optimal viewpoint in an automatic fashion. It should be noted that manual camera control is a common application mode in clinical practice, while autonomous camera mode is a tentative control mode and it is not currently used in clinical practice for the safety purpose.

**Fig. 1:**
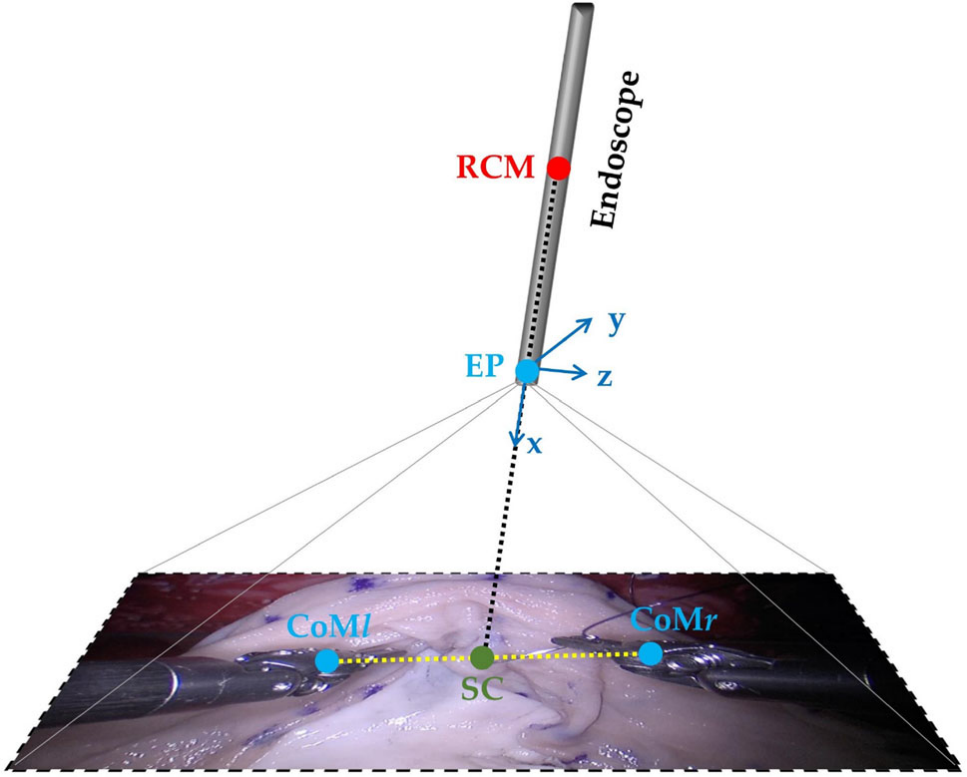
Perspective representation of how the position of the endoscope in Cartesian space is changed according to the position of the instruments

$\bullet$ Manual camera control (MC) modality: the endoscope position is handled in the traditional way, i.e., with the dedicated foot pedal on the master console, it is possible to suspend PSMs’ teleoperation and switch to endoscope control.

$\bullet$ Autonomous camera control (AC) modality: the system continuously tracks the surgical tools and accordingly adjusts the endoscope’s position (EP), resulting in an ongoing adjustment of the field of view (FoV). For example, the FoV can be centered on the midpoint of the centers of mass of the two surgical tools (CoMl and CoMr for the left and right tool respectively). As shown in Fig. 1, *SC* means the scene center, and RCM stands for remote center of motion that in MIS corresponds to the small incision through which the surgical tools or the camera penetrate the patient’s skin [13].

**Fig. 2:**
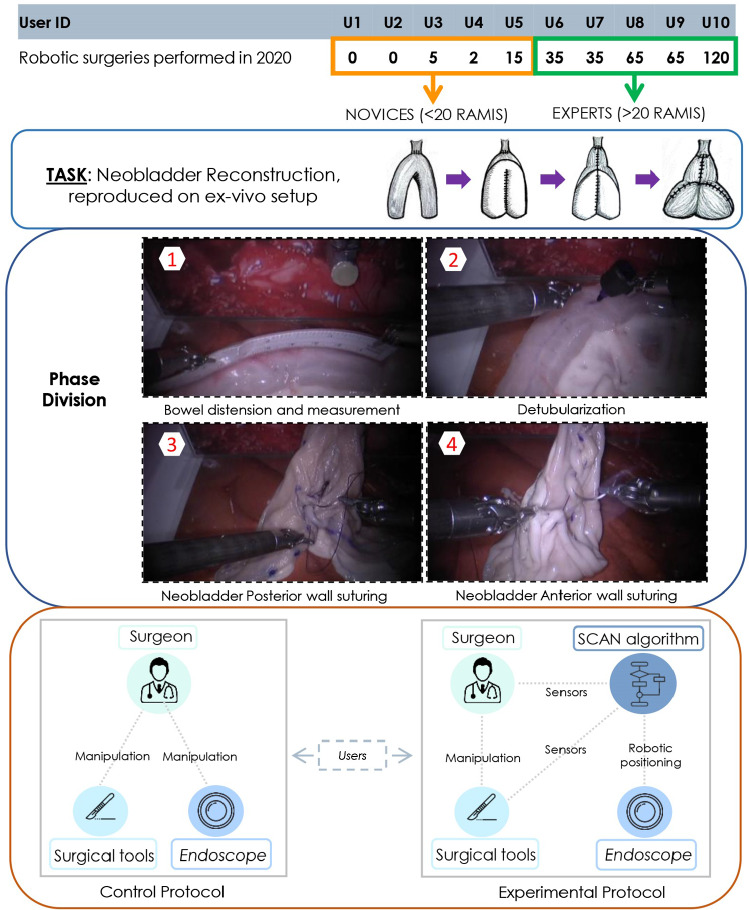
This figure shows the details of the surgical protocol. Ten surgeons were divided into novices and experts according to their surgical experience. Then, the users were requested to perform the simplified Neobladder Reconstruction twice randomly to compare the two different endoscopic control modalities

**Fig. 3:**
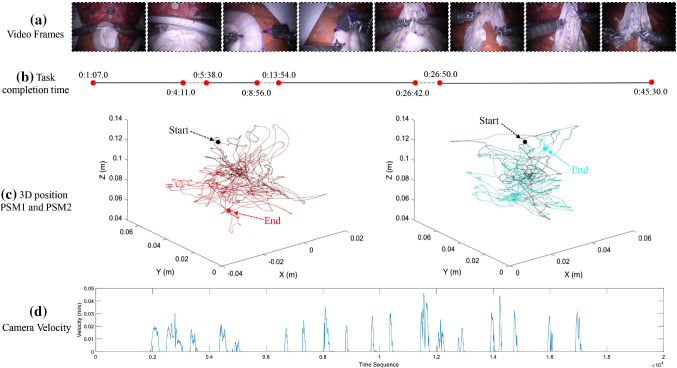
One sample of the raw data (User 6, control protocol). (a) shows some video frames in this Neobladder Reconstruction, and (b) is the task completion time divided by four phases. (c) is the 3D position of PSM1 and PSM2, the last one (d) denotes the camera velocity. The display of PSMs position and camera velocity is only partially shown to avoid congestion caused by too many data points

### Surgical protocol

Twelve surgeons from the European Institute of Oncology (Milan, Italy) were requested to perform an orthotropic neobladder reconstruction, and data from 10 participants are analyzed for this skill evaluation. (The remaining two surgeons failed to perform this procedure caused by external factors, so this part of data was discarded). Since the users were characterized by a different level of robotic manipulation experience, they were divided into two groups: participants who had performed less than 20 robot-assisted MIS operations were considered novices, while those who performed more than 20 were deemed experts. For the experiment, this surgical procedure was reproduced on an ex vivo porcine model in a simplified version, with the aim of minimizing the waste of animal tissue. Three steps were selected to be reproduced: (i) bowel distension and measurement; (ii) detubularization; (iii) neobladder plication and suturing [14]. Each surgeon was asked to perform the simplified neobladder reconstruction twice, randomly starting with MC or AC. This configuration is to reduce the prior knowledge and memory when users repeat the same experiment for a short time. Figure 2 shows the details about this protocol. The control protocol means that the user completes the experiment by manually manipulating the endoscope, while the experimental protocol is accompanied by the assistance of autonomous navigation of the endoscope.

### Data acquisition and preprocessing

At the end of a da Vinci robot operation session, the system makes available two different types of data that have been successfully exploited for this analysis: system data and video data. System data include all the information made public by the dVRK system, such as kinematic variables (e.g., poses, twists, wrenches), joint space variables (e.g., angular velocities, torques), system information and sensor data. On the other hand, video data include compressed images recorded by the endoscope. Figure 3 demonstrates a sample of the collected raw data.

Two main preprocessing steps have been carried out. The first one was “phase division” and to perform it endoscopic video data were exploited. On the basis of the advice and opinions taken from expert surgeons involved in the experiment, the entire surgical procedure was divided into four distinct sub-phases: (1) bowel distension and measurement; (2) detubularization (simulated with the demographic pen); (3) neobladder posterior wall suturing; (4) neobladder anterior wall suturing. It was considered appropriate to treat the suturing of the walls in two separate phases because, according to the expert’s opinion, the two tasks are considered to have different difficulty levels. In particular, anterior wall suturing is deemed to be more complex with respect to posterior wall. Here, the phase division of all data was completed by an annotator under the supervision of the expert. The second step was to carry out a preliminary cleaning of the data to eliminate inconsistencies and prepare it for the following analysis. This is done to highlight whether, throughout the entire duration of the operation, there were any interruptions to the workflow and then to cut out from the signal all the parts where the surgeon was not actually performing the task. To accomplish this, the data provided by the 4 head sensors placed on the HRSV located at the master console proved to be available. These sensors provide messages that can take on 3 possible values: 0, 1 or 2. When the data is 0, this is interpreted as an interruption of the operation since it indicates that the sensor has detected surgeon’s head detachment. If such sensors reveal the occurrence of an interruption, the kinematic data corresponding to the time intervals would be discarded.

### Metrics for surgical skill evaluation

To evaluate the users’ performance in executing the experiment, some metrics [15, 16] for surgical skill evaluation have been defined. Each metric will assume a different value for each user $i \in [1,10]$, for each phase $j \in [1,4]$, and for each control strategy $k=$ “*e*”, “*c*”, where “*e*” means the experimental protocol, “*c*” is the control protocol. Here, experimental and control protocols represent the operation in AC and MC, respectively.

#### Time-related metrics

Task completion time (CompTime) is defined as the total time (in seconds) required to complete the task:

1 $$\begin{aligned} \text{ CompTime } _{i, j, k}=t_{\text{ end } }-t_{\text{ start } } \end{aligned}$$

where $t_{\text{ end }}$ and $t_{\text{ start }}$ are the phase ending and starting time instants, respectively. A shorter CompTime means the task is completed in less time. Economy of motion (EoM) is defined as the total length (in meters) of the curve described by the tip of the instruments in space from the beginning until the completion of the task [17], and a smaller EoM denotes the operation is performed with less useless instrument movement. The total path length traveled by the tools was computed with the following formula:

2 $$\begin{aligned} P L_{i, j, k}=\int _{t_{\text{ start } }}^{t_{\text{ end } }} \sqrt{\left( \frac{d x}{d t}\right) ^{2}+\left( \frac{d y}{d t}\right) ^{2}+\left( \frac{d z}{d t}\right) ^{2}} d t \end{aligned}$$

where *x*, *y* and *z* represent the Cartesian coordinates of the position of the instrument (PSM1 or PSM2) in the 3D space at time instant *t*. The final value of the metric is then obtained by summing what results from applying the previous formula to the kinematic data of both the PSMs:

3 $$\begin{aligned} {\text {EoM}}_{i, j, k}=P L_{P S M 1}+P L_{P S M 2} \end{aligned}$$

#### Camera movement-related metrics

Camera metrics are related to the endoscope’s movement. To compute them, ECM velocity data are exploited in order to define endoscope motion or stillness. More precisely, an endoscope movement is considered to begin when the velocity is nonzero and to end when the velocity returns to zero. The time intervals of endoscope motion are then computed, and a certain number of durations (in seconds) are thus obtained: $\{T_{1}, T_{2}, \ldots , T_{N}\}$. Camera movement frequency (CFreq) is defined as the average number of endoscope movements made by a surgeon over the entire exercise:

4 $$\begin{aligned} \text{ CFreq } _{i, j, k}=\frac{N}{T_{t o t}} \end{aligned}$$

where *N* represents the number of movements performed by the endoscope in the considered phase, $T_{tot}$ is the total duration of the chosen phase. The larger CFreq means the higher camera movement frequency for the optimal surgical field of vision. Camera movement duration (CDur) is defined as the average time in seconds of all endoscope movements over the entire exercise, and the shorter CDur denotes that surgeons spend less time adjusting the viewpoint of the endoscope:

5 $$\begin{aligned} \text{ CDur } _{i, j, k}=\frac{1}{N} \sum _{t=1}^{N} T_{t} \end{aligned}$$

#### Performance metrics

Similar as [15], the overall performance metrics $P_{i, j, k}^{\text{ time-accuracy } }$ and $P_{i, j, k}^{\text{ camera } }$ were defined for both time-related metrics and camera movement-related metrics. Given the considerable variability of the data collected due to the different experience of the surgeons involved and the different strategies that they used, it was considered appropriate to first carry out normalization of the metrics so that they could take on values between 0 and 1:

6 $$\begin{aligned} \overline{m_{i}}=\frac{m_{i}-m_{min}}{m_{max}-m_{min}} \end{aligned}$$

where $\overline{m_{i}}$ represents the normalized value of the metric, ${m_{min}}$ and ${m_{max}}$, respectively, represent the lowest and the highest value that the metric being considered assumes among all its realizations. Performance metrics are thus computed:

7 $$\begin{aligned} P_{i, j, k}^{\text{ time-accuracy } }=\frac{(1-\overline{ \text{ CompTime } })+(1-\overline{ \text{ EoM } })}{2} \end{aligned}$$

8 $$\begin{aligned} P_{i, j, k}^{\text{ camera } }=\frac{\overline{ \text{ CFreq } }+(1-\overline{ \text{ CDur } })}{2} \end{aligned}$$

**Fig. 4:**
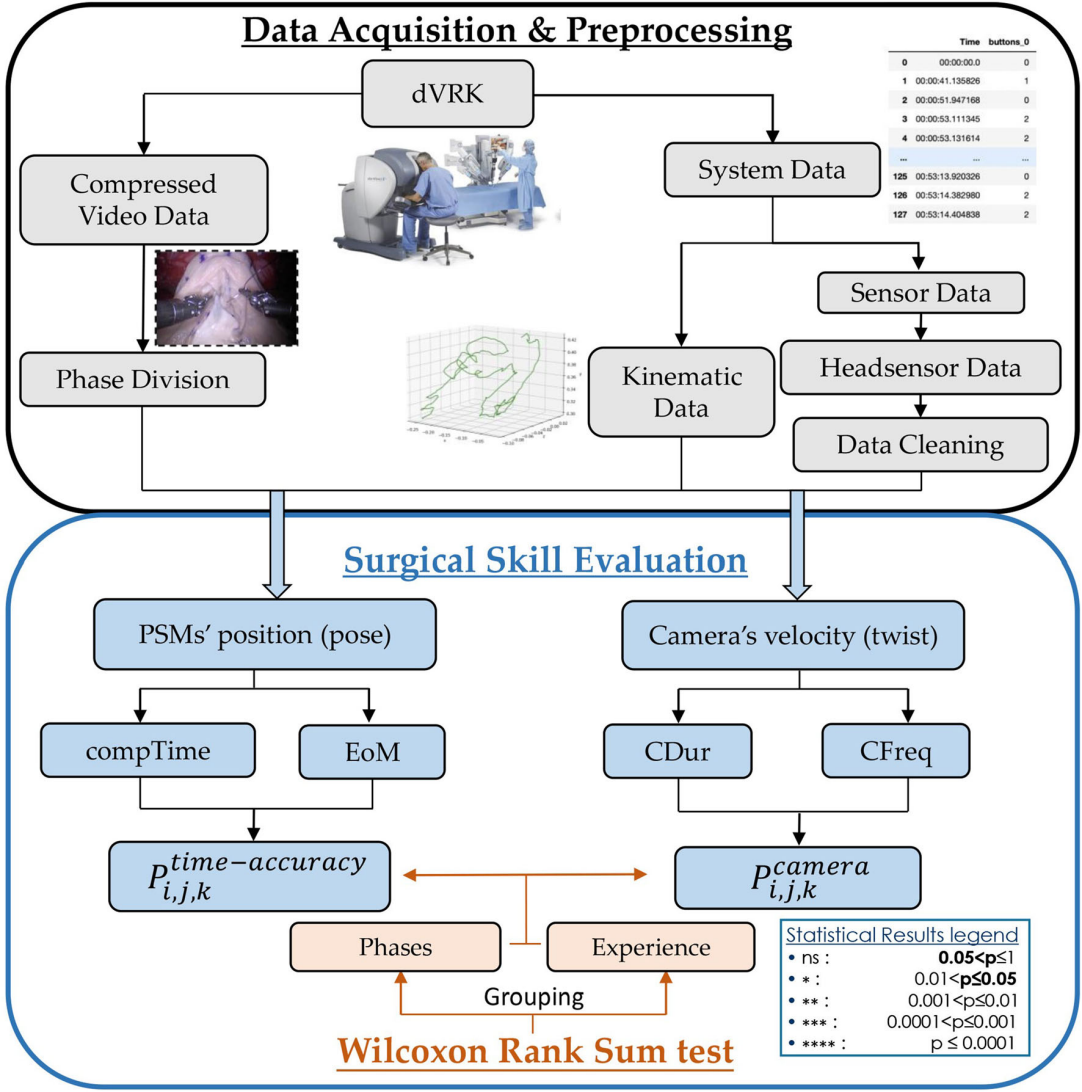
Workflow diagram of the data analysis. The above block explains the necessary data, including video and system data, while the following block shows the adopted surgical skill assessment approaches in our surgical skill evaluation

where the upper bar stands for normalized metric, obtained by applying (6). A larger $P_{i, j, k}^{\text{ time-accuracy } }$ means that the surgeon has a better performance in operating the surgical instruments to complete the entire surgical task, since it presents that the surgeon can perform the whole task with less useless movement of the instruments in less time. For the metric of $P_{i, j, k}^{\text{ camera } }$, a larger value shows that the surgeon can better control the movement of the endoscope to maintain the optimal surgical field, which is related to the surgical safety. It should be noted that the control of the endoscope and the surgical instruments is separated in the manual control mode of the endoscope, while the movement of the endoscope follows the position of the surgical instrument in the autonomous mode.

**Fig. 5:**
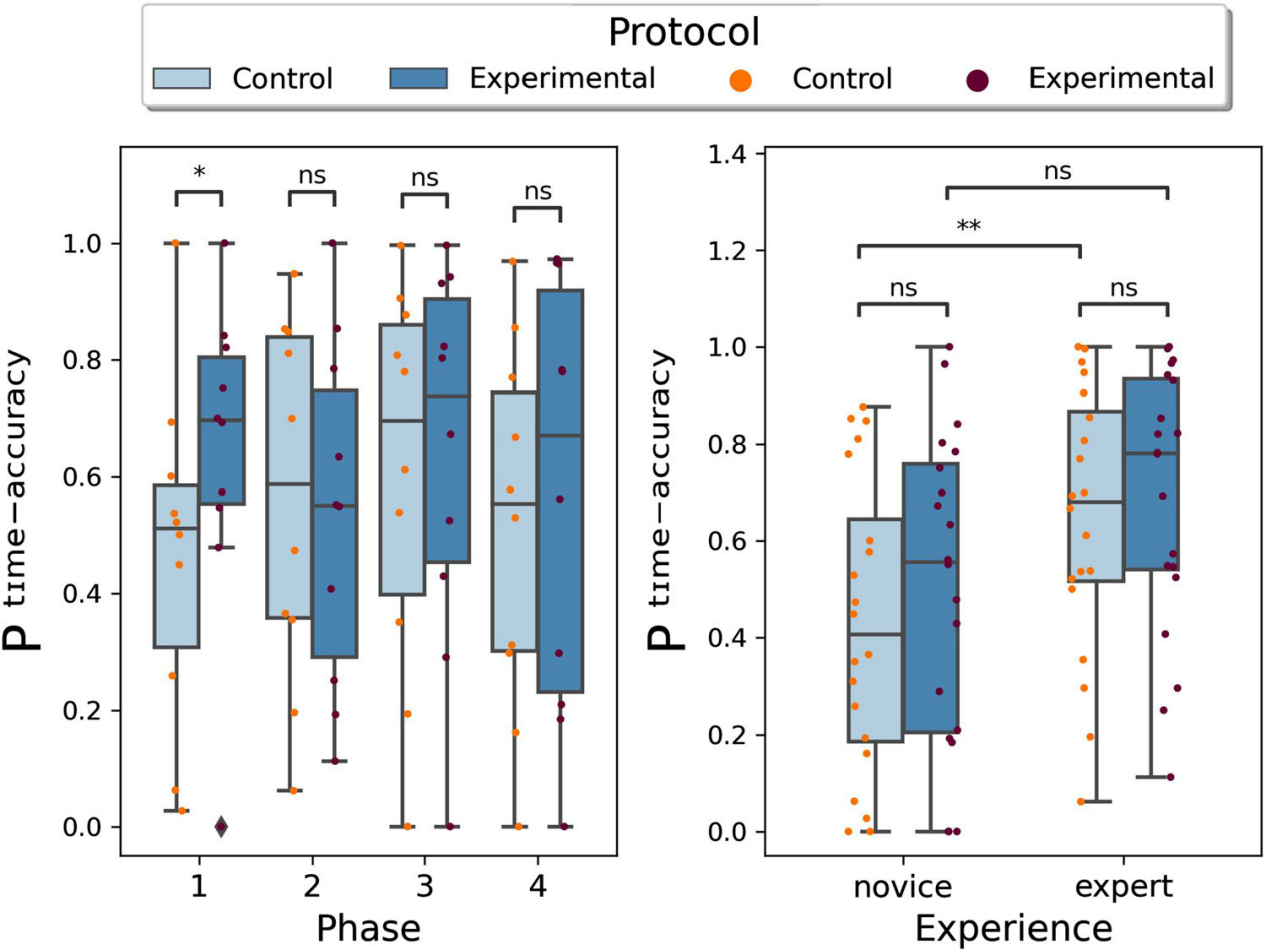
Box plots of $P^{\text{ time-accuracy }}$. Control protocol means the users with manual control, while experimental protocol is with autonomous control. Different protocol comparison grouping results according to different phases (left) and different experience (right). In the left figure, phase 1 is bowel distension and measurement, phase 2 represents detubularization, phase 3 denotes neobladder posterior wall suturing, and phase 4 is neobladder anterior wall suturing

**Fig. 6:**
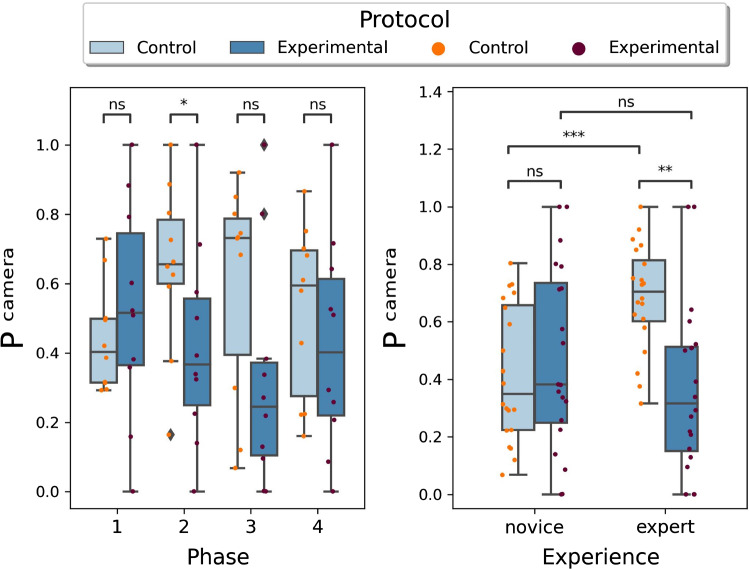
Box plots of $P^{\text{ camera }}$. Different protocol comparison grouping results according to different phases (left) and different experience (right). In the left figure, phase 1 is bowel distension and measurement, phase 2 represents detubularization, phase 3 denotes neobladder posterior wall suturing, and phase 4 is neobladder anterior wall suturing

### Statistical analysis

Due to the relatively small sample size, nonparametric statistical significance tests were exploited to compare the objective metrics between the different camera control modalities. Specifically, the Wilcoxon rank sum test [18] was used, considering differences to be statistically significant when $\mathrm { p} \le 0.05$. Once the metrics have been computed, outcomes are evaluated in pairs according to different criteria. Metrics relative to the same phase are firstly compared when considering different control strategies. Subsequently, users are grouped according to their experience and the statistical analysis is applied both between participants of different experience considering the same protocol and then differences are searched considering the outcomes of different protocols for novices and experts separately. The main objective is to investigate what influence both experience and the method of endoscope control can have on the performance of surgeons. Figure 4 presents a workflow diagram of the framework in which this analysis has been developed, starting from the collection and preprocessing of the data up to the goal of surgical skill evaluation.

## Results and discussion

This analysis is conducted to investigate whether there are significant differences between the metrics that result when considering the control configuration (MC) and those considering the experimental case (AC). Also, it is possible to group the obtained data in different ways to highlight different features, if any.

Figures 5 and  6 show the box plots of Performance metrics $$P^{\text{ time-accuracy }}$$ and $$P^{\text{ camera }}$$, respectively. The bars represented on top serve to show the pair of data to which Wilcoxon’s paired analysis was applied. The resulting *p*-value is associated to a certain label, depending on the significance of the result, according to the following legend: (i) ns: $$0.05<\mathrm { p} \le 1$$; (ii) $$ * $$: $$0.01<\mathrm { p} \le 0.05$$; (iii) $$**$$: $$0.001<\mathrm { p} \le 0.01$$; (iv) $$***$$: $$0.0001<\mathrm { p} \le 0.001$$; (v) $$***~*$$: $$\mathrm { p} \le 0.0001$$.

Figure 5 left shows a significant difference ($$*$$) of the value assumed by the metric in phase 1 ($$p=0.0284$$). Grouping the results according to experience (Fig. 5 right) shows a significant difference ($$**$$) in the performance achieved by novice and experts during the control protocol ($$p=0.003654$$).

On the other hand, Fig. 6 left shows a significant difference ($$*$$) in phase 2 in terms of camera performance ($$p=0.01953$$). Considering the experience-based division, represented in Fig. 6 right, it shows a significant difference ($$***$$) during the control protocol ($$p=0.0004826$$), and a difference ($$**$$) between expert surgeons executing the task with the 2 different control modalities ($$p=0.003153$$). In particular, the performance appears to decrease when autonomous endoscope control rather than manual control is used.

It can be stated that $P^{\text {time-accuracy}}$ metric proved useful in highlighting significant differences between control and experimental protocols, but only in phase 1. In particular, the performance appears to increase when autonomous endoscope control rather than manual control is employed. The reason for this can be attributed to the fact that, according to the way the sub-phases of the total task have been defined, it is in the initial phases that the movement of the endoscope must be more frequent and extensive. In particular, the measurement operation requires a frequent change of viewpoint, more than what is needed to perform, for example, the suturing task. It shows that if the surgeons need to perform some surgical operations with frequent field switching, the autonomous movement of the endoscope can improve the performance of surgeons in operating the surgical instruments, while the different control modes of the endoscope do not introduce significant difference in the operation of surgical instruments if the visual field is relatively fixed. In addition, this metric was also useful in highlighting the different experience of the surgeons. Overall, it results in higher values for experienced surgeons if compared to the case of novice surgeons. It also presents the autonomous movement of the endoscope can narrow the difference between novices and experts in the manipulation of surgical instruments, since the significant difference between novices and experts in the manual control disappears when the endoscope moves autonomously. Nevertheless, novices need more practice in the operation of surgical instruments, since they got lower values in both modes of the endoscope compared with experts.

For the metric $P^{\text{ camera }}$, it proved useful in highlighting significant differences between control and experimental protocols, but only in phase 2. It can be seen that the autonomous movement of the endoscope reduces the value of the camera metric if the surgical field of vision is relatively fixed, so it is easier for surgeons to manually control the endoscope to maintain the best surgical field of vision in this case. Moreover, a decrease in the score achieved by experienced surgeons can be noted, evidenced by a significant difference between the results obtained in the two control mode cases. This can be attributed to the greater cognitive load that a subject already accustomed to and experienced in operating the endoscope in the manual configuration is subjected to when having to learn a new control strategy, even if it is automatic. Hence, experts need more time to adapt to the new control strategy compared with novices. Furthermore, there is a significant difference between novices and experts in the manual operation mode, which indicates that novices do not make full use of endoscopes in the operation process. In other words, they do not maintain the optimal surgical vision compared with experts, so novices need to practice their ability to manually operate endoscopes in order to keep the optimal surgical vision.

After analyzing the experimental data, four findings can also be summarized to improve the performance of surgeons: (1) The autonomous movement of endoscope is more suitable for the operation that needs frequent switching of surgical viewpoint, while it does not bring great improvement when the visual field of surgical task is relatively fixed. (2) The manipulation ability of novices for surgical instruments is always lower than that of experts under different control modes, which means that novices need to strengthen this practice in the operation of instruments. (3) The automatic motion mode of endoscope is a challenge for experts due to their prior knowledge, so they need more time to adapt to the new mode. (4) The endoscope does not maintain the optimal surgical vision in the manual operation mode for the novices compared with experts, so the novices need to improve the manipulation ability of the endoscope for the surgical safety.

## Conclusions

This paper conducted a quantitative evaluation of surgical skill based on the objective criteria involving surgical instruments and endoscope. An ex vivo neobladder reconstruction was performed by 10 urologists using SCAN framework or manual endoscope control randomly. Two different normalized metrics were used to evaluate surgical performance, and it showed promising results for the skill evaluation and provided the practical guidance for surgeons. The evaluation strategy in this paper uses various accessible data in robotic surgery, including videos, robot kinematics, sensor data, etc., and considers the movements of both surgical instruments and the endoscope, which enhances its generalization and potential in other robot assisted surgeries.

One notable point is that the division standard for novices and experts in this paper is under the agreement of an expert, in order to balance the distribution of novices and experts. More factors, such as the learning curve, will be considered in future work to better divide surgeons at different levels. Furthermore, users who repeat the experiments in a limited time may be affected by muscle memory and prior knowledge, which means that the data of usability study may need to be further expanded. More surgical data will be collected to verify the versatility of the proposed approach in the future.
